# Reward Modality and Task Complexity Shape Learning Dynamics during Multi-decision Sequential Navigation

**DOI:** 10.1523/ENEURO.0126-26.2026

**Published:** 2026-09-18

**Authors:** Juliette Archimbaud, Charlotte Lehericy, Céline Auger, Christelle Rochefort, Laure Rondi-Reig

**Affiliations:** ^1^CeZaMe lab, Center of Neuroscience Neuro-SU, Inserm, CNRS, Sorbonne Université, Paris F-75005, France; ^2^CeZaMe lab, IBPS, Institut de Biologie Paris-Seine, Inserm, CNRS, Sorbonne Université, Paris F-75005, France; ^3^Cerebellar Neurophysiology, CNRS UMR 8003 - Saints-Pères Paris Institute for the Neurosciences, Paris F-75006, France

**Keywords:** goal-directed navigation, MFB stimulation, reward, sequence

## Abstract

Learning dynamics depend on internal and contextual multifactors among which the interaction between motivational context and task demands plays an important role. Spatial navigation provides a powerful framework to study these interactions as it integrates cognitive, motivational, and motor components. To examine how reward modality and task complexity modulate learning dynamics in male mice, we used a sequential navigation task in which the number of decision points progressively increased across training, while preserving the same geometrical context and goal location. We compared three versions of the same task, in which the fixed goal location was rewarded by either escape from water (aquatic version), food pellets, or medial forebrain bundle (MFB) stimulation associated with different levels of complexity. At low task complexity, the aquatic group achieved a higher level of performance. However, this effect does not extend to the acquisition of the most complex sequence, as all groups ultimately converge toward comparable abilities to perform direct trials. Furthermore, reward type appears to modulate motivational engagement and speed in all training conditions. Together, these data show that, during spatial navigation, reward modality influences not only the level of learning but also its behavioral execution.

## Significance Statement

Navigation relies on the interaction between cognitive demands and motivational context, yet their combined influence on learning dynamics remains poorly understood. Here, we compare how different reward modalities shape the acquisition of a sequential navigation task within a single, controlled environment, while systematically increasing task complexity. We show that reward modality strongly biases early strategy selection under low cognitive demand and continues to modulate learning acquisition as the number of successive choices increases. By isolating reward effects within the same behavioral structure, this work provides an insight into understanding how motivation and task complexity jointly shape navigation strategies.

## Introduction

Spatial navigation refers to the ability to move through and orient within the environment. This goal-directed behavior relies on the flexible use of allocentric (world-centered) and egocentric (body-centered) representations, as well as on strategy selection shaped by task demands and sensory context (Trullier and Meyer, 1997; Arleo and Rondi-Reig, 2007; Vijayabaskaran et al., 2025). Early theoretical frameworks emphasized the hippocampal cognitive map as a substrate for allocentric navigation, integrating landmark and self-motion information to build a coherent spatial model (O’Keefe and Nadel, 1978; Gallistel, 1990). Subsequent work has shown that successful navigation also depends on route-based and sequential processes, particularly in tasks involving repeated and visually identical decision points (Thinus-Blanc, 1996; Rondi-Reig et al., 2006; Iglói et al., 2009). In such contexts, performance depends less on global map construction than on the accurate acquisition and retrieval of ordered action sequences. Accordingly, hippocampal representations can be viewed not only as spatial maps but also as mechanisms linking stimuli and experiences across spatial and temporal dimensions (Eichenbaum and Cohen, 2014). Sequential navigation therefore places specific demands on memory systems as animals must link successive choice points to coherent actions while maintaining task engagement across trials and days. Previous work has shown that hippocampal and striatal systems contribute differentially to place-based, response-based, and sequence-based learning (Packard and McGaugh, 1996; Fouquet et al., 2013), and strategy selection may shift as tasks become automatized or more cognitively demanding.

Yet, learning dynamics cannot be fully understood without considering motivational context. Navigation does not depend solely on spatial memory but also on how rewards shape engagement and persistence. Animals must associate their actions with expected outcomes while initiating and sustaining goal-directed movement toward those outcomes. Dopaminergic reward prediction error signals shape action–outcome learning and guide adaptive choice behavior (Schultz et al., 1997; Schultz, 2015), while neural systems computing subjective value integrate expected reward, effort, and uncertainty (Shizgal, 1997; Arvanitogiannis and Shizgal, 2008). Navigation thus emerges from the interaction between memory systems encoding spatial information and reward-related circuits evaluating the value of actions. Within this framework, navigational choices such as selecting one route over another at an intersection can be understood as value-based decisions in which behavioral outcomes are weighted by expected reward and associated costs.

To investigate how distinct reward contexts influence learning dynamics and behavioral strategy during sequential navigation, we took advantage of the flexible design of the Starmaze (Rondi-Reig et al., 2006; Jarlier et al., 2013). We created three versions of the same task, each using a different reward modality delivered at the same goal location: (1) escape from water in the aquatic Starmaze, (2) food reward in the dry Starmaze, and (3) medial forebrain bundle (MFB) stimulation. Task difficulty was progressively increased by varying the number of required decision points, from a direct path with no choice to sequences requiring one, two, or three correct turns to reach the goal. Increasing the number of intersections augments sequential memory load and decision complexity, requiring the animals to maintain reward representations across successive actions. This design therefore enables a controlled comparison of how reward modality interacts with cognitive demand while spatial structure and goal location remain constant. By analyzing performance, motivational indices, and fine-scale trajectory dynamics across increasing levels of task complexity, we examined how distinct reward contexts interact with cognitive demand to shape learning dynamics and behavioral strategy during navigation.

## Materials and Methods

### Experimental animals

Thirty-four male C57BL/6J mice (8–14 weeks old, Janvier Labs) were used in this study. They were all trained in the Starmaze navigation task but distributed in three groups based on the modality of reward: in Group 1 (*n* = 14), mice were trained in an aquatic version of the Starmaze in which rewards consisted of a safe escape on a dry platform; in Group 2 (*n* = 14), mice were food-deprived and rewards consisted of food pellets delivered through a food dispenser; and in Group 3 (*n* = 6) rewards were delivered through MFB stimulation. Mice were housed in groups of up to five animals under standard conditions: 12 h light/dark cycle, with *ad libitum* access to water and food. Seven days prior to the beginning of behavioral tests, mice were isolated and housed in an animal facility in individual cages with access to enrichment (nesting material and a toy), and food deprivation was performed in Group 2. All behavioral experiments took place during the light cycle and were conducted in accordance with the European Union Directive (Directive 2010/63/EU) regarding the care and use of animals for experimental procedures in compliance with the Ministère de l’Agriculture et de la Forêt, Service Vétérinaire de la Santé et de la Protection Animale (#28025-2020111721361293 v8) of the Ministère de l'enseignement supérieur, de la recherche et de l'innovation.

To assess potential motor deficits following implantation, all three groups' motor abilities were tested using a static balance and a motor coordination task.

### Unstable platform

The static balance of mice was assessed using a 9 cm circular platform fixed on a 105 cm vertical rod. The platform tilted in all directions according to the position of the mouse. The holding time is recorded during three trials of a maximum of 3 min. If the mice fell before 20 s, they were immediately returned to the platform.

### Accelerated rotarod

Motor coordination was assessed using a rotating horizontal cylinder (44 cm height). Mouse holding time was recorded during three trials. Each trial lasted a maximum of 5 min, during which the speed was gradually increased from 4 to 40 rpm, with an increment of 1 rpm every 8 s.

### MFB implantation

Group 3 mice were implanted under deep isoflurane anesthesia with bipolar stimulation electrodes (two 150-µm-diameter stainless steel electrodes twisted together; Plastics One) targeting the left MFB (AP +1.4, ML +1.2, DV +4.8). After surgery, mice were allowed a minimum of 1 week of recovery before behavioral assays.

### MFB functional implantation testing

To check for functional implantation of the MFB stimulating electrode, Group 3 mice were tested in a place preference task using a nontransparent 31 × 24 × 40 cm box with two 2 cm diameter holes on the same wall (Extended Data Fig. 1-1). Exploration of the left hole was automatically detected by Smart Tracking software and was rewarded with an MFB electrical stimulation, while there was no consequence for exploring the right hole. Each stimulation consisted of a 140 Hz train of negative pulses lasting 100 ms. Stimulation intensity was incrementally increased from 0 to 40 µA, and two trials of 180 s for each intensity were performed over 2 d (Extended Data Fig. 1-1). For each trial, both the number of entries into the rewarded compartment and the time spent in the rewarded compartment were quantified. Because stimulation was delivered upon each entry into the rewarded zone, animals had to leave and re-enter the compartment to trigger additional stimulation trains. Consequently, the number of entries may reflect not only place preference but also the animal's learning that repeated exits and re-entries are required to obtain additional rewards. MFB implantation was considered successful when mice reached a number of entries in the rewarded zone greater than twice the mean number of entries at 0 µA. The optimal intensity ranged from 30 to 40 µA, depending on individual mice.

### Food deprivation

Before starting the training procedure in the Starmaze, Group 2 mice were food-restricted for 7 consecutive days. During this period, 2–4 g of food was delivered daily to each mouse in order to maintain the weight loss below 15% of the mouse's initial weight.

### Sequential navigation in the Starmaze

**Figure 1.**
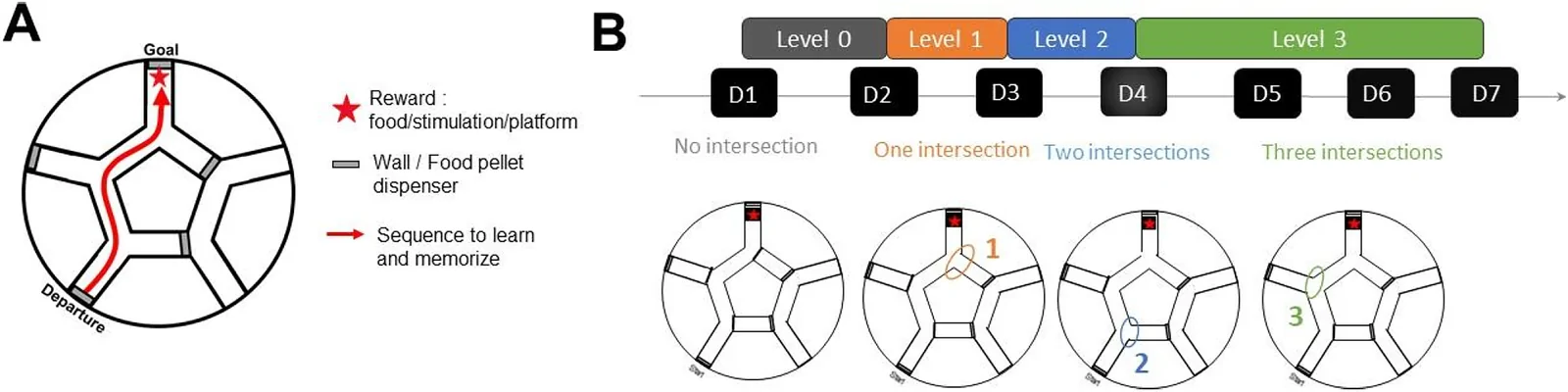
Diagram of the experimental setup. ***A***, Mice are trained in the Starmaze, a maze with five peripheral alleys. Training is conducted progressively, with intersections numbered 1, 2, and 3 being opened incrementally from Day 1 to Day 4. The sequence to be acquired is represented in red. Three groups of mice, each receiving a distinct type of reward (indicated by a red star), are involved in the experiment: a food reward, intracranial stimulation of the medial forebrain bundle (MFB), or access to a platform allowing the mice to escape from water. ***B***, The protocol lasts for 7 d (D1 to D7) and includes four levels of difficulty, denoted by numbers (0, 1, 2, and 3), which correspond to the number of peripheral alleys that are opened. Each day consists of four sessions, and each session comprises four trials.

In all mice, a sequence-based, reward-oriented navigation task was performed in the Starmaze composed of a central stem from which two Y-mazes originate (Fig. 1). External and internal alleys were 40 and 24 cm long, respectively, and both were 18 cm wide. Low light intensity was maintained ∼10–20 lux. White noise (50 dB) was broadcast in the testing facility to cover auditory cues that the mice could have used to orient themselves. A circular black curtain was placed around the maze so that the mice could not rely on visual cues to learn and memorize the path. For the aquatic Starmaze, alleys were filled with water made opaque with an inert nontoxic product (Accuscan OP 301, Brenntag) to hide a platform 1 cm below the surface in the aquatic version. Water temperature was maintained between 18 and 20°C.

On each trial, mice had to reach a goal zone from a departure point to receive a reward consisting either of MFB stimulation (three trains of 100 ms at 140 Hz, 2 s apart; Starmaze with MFB), three food pellets (food reward Starmaze), or water escape by setting on a dry platform (aquatic Starmaze). The current intensity of the MFB stimulation was set between 20 and 30 µA at the beginning but could be adjusted progressively to up to 50 µA depending on the motivation of the mouse. The departure point and the goal zone were fixed so that the shortest way to reach the reward was to execute a left-right-left sequence of turns. Each trial lasted at most 90 s, with a 1 min intertrial period, during which the maze was cleaned using 20% ethanol in the dry Starmaze versions. The animal repeated four such trials within a session, and four sessions were performed per day, with a 30 min intersession period. This schedule was carried out for up to 7 d. As all three intersections of the maze were visually identical, mice had to remember the temporal sequence of turns to solve the task. Mice were trained using an incremental protocol in which task complexity increased across three levels. At each level, animals completed full daily sessions under stable conditions, followed by an additional session on the next day before task demands were increased. The highest level of complexity was maintained for 4 d. Learning was assessed using an operational criterion of 75% direct trajectories per training day. This incremental protocol was implemented through a structured pretraining phase in which maze configuration and task demands were progressively adjusted across sessions. During the first three and a half days, mice were “pretrained” in a progressively more complex maze configuration. During the first day, part of the alleys between the departure point and the goal location were closed, so the mice could only use one path to get to the reward. The following days (2, 3, and 4), alleys were progressively opened one after the other (Fig. 1) . To consolidate the learning of the task, the Starmaze configuration was the same during the first two sessions of each day as during the last two sessions of the previous day. For each session, the percentage of direct trials, latency to reward, latency to exit the departure alley, and the mean speed were measured.

### Tissue processing and histological procedures

Following the final round of behavioral tests, animals in the MFB group were deeply anesthetized with an overdose of anesthetics (xylazine 20 mg/kg/ketamine 200 mg/kg, administered intraperitoneally) and perfused intracardially with saline, followed by 4% paraformaldehyde (PFA) in a 0.1 M phosphate buffer (PB). The brains were removed, postfixed for 2–3 d in 4% PFA, and then stored at 4°C in 0.1 M PB with 0.02% sodium azide. For histological verification of the placement of the MFB electrode (Extended Data Fig. 1-3), the brains were embedded in 3% agarose and then sliced into 100-μm-thick coronal sections. These sections were mounted on slides and coverslipped with a mounting medium containing DAPI (Fluoromount-G with DAPI, Fisher Scientific). Images were taken using an Axiozoom v16 microscope (Carl Zeiss) equipped with a 2.3× objective. The tip of the electrode tracks was identified by localized mechanical lesions in the MFB according to a stereotaxic atlas. 

### Data acquisition and statistical analysis

Data acquisition was performed by means of a video recording system and the tracking software ANY-maze. Statistical analyses were run using GraphPad Prism version 9. Normality was tested using the Shapiro–Wilk test. An ordinary one-way ANOVA was performed for accelerated rotarod analysis. The Kruskal–Wallis test was performed for static balance analysis. Two-way repeated-measures ANOVAs were performed for Starmaze performance analysis. The significance level was set at 5%.

## Result

Mice performances were evaluated using three different types of reward and following an incremental training protocol divided into three levels of difficulty (Fig. 1; see Materials and Methods). Depending on the version of the task, mice had to reach the goal zone to be rewarded either with MFB stimulation (stimulation group, *n* = 6), food (food group, *n* = 14), or with the ability to escape from water on a dry platform (platform group, *n* = 14). Several parameters were used to evaluate willingness to engage in the task, performance, motivation, as well as path optimization across the different levels of difficulty.

10.1523/ENEURO.0126-26.2026.f1-1 Figure 1-1 **Functional validation of the MFB electrode implantation in the place preference task. A.** A train of MFB stimulation pulses was delivered upon entrance in the rewarded zone (red). **B.** Place preference was assessed over two consecutive days using increasing stimulation intensities ranging from 0 to 40 µA (two consecutive 180-s trials at each intensity). Three mice already exhibited robust place preference at 30 µA on Day 2 and were therefore not tested at 40 µA. **C.** Number of entries into the rewarded zone as a function of current intensity for the 6 individual mice included in the MFB group (M1 to M6). **D)** Time spent in the rewarded zone as a function of current intensity for the 6 individual mice included in the MFB group (M1 to M6). The dashed line indicates the level of exploration in the absence of stimulation. Grey and black dots correspond to the first and second trial of each day, respectively. Download Figure 1-1, TIF file.

**Figure 2.**
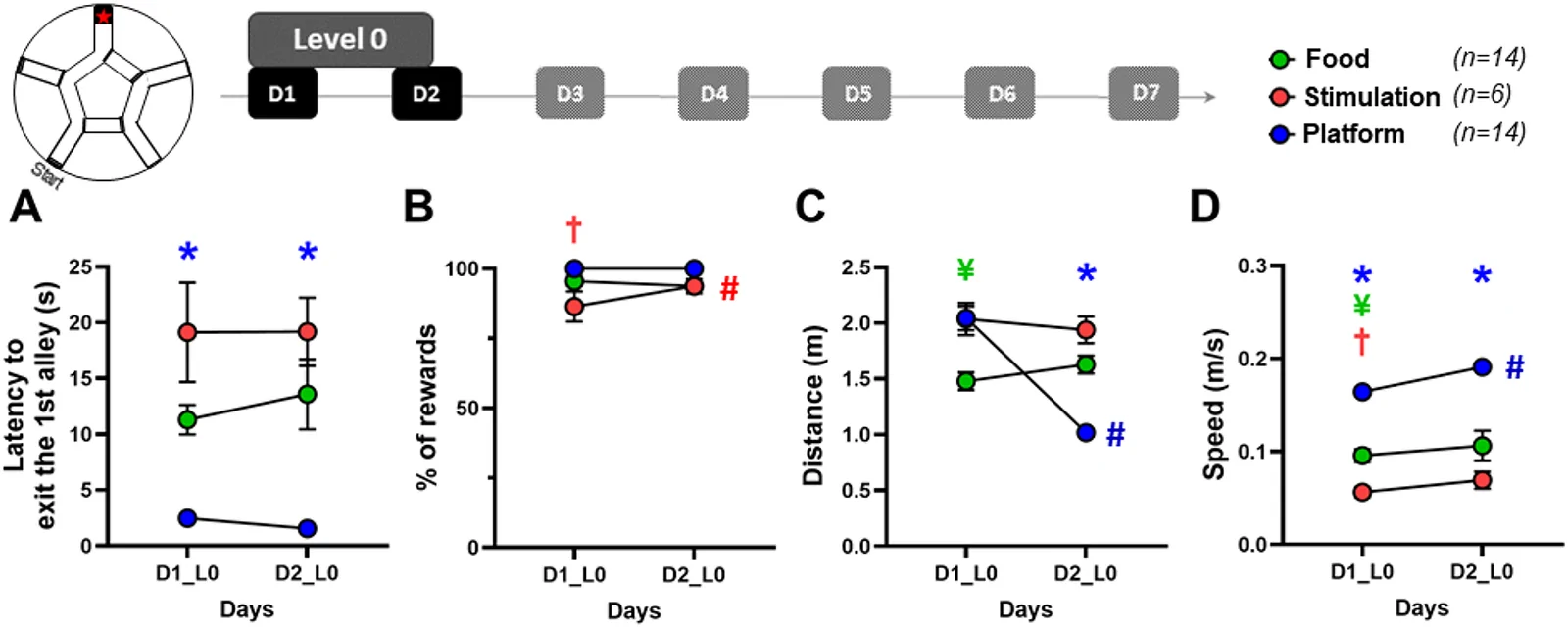
Goal-directed sequence with no decision point (Level 0, “no intersection”). This figure illustrates the performances of the three groups (platform, stimulation, and food) compared in terms of (***A***) latency to exit the first alley of the Starmaze; (***B***) percentage of trials in which mice get the reward; (***C***) distance covered to reach the reward; and (***D***) the mean speed. *the platform group is different from the two other groups. ^†^the stimulation group is different from the two other groups*.* ^¥^the food group is different from the two other groups. ^#^represents a significant day effect for the color-associated group (Extended Data Fig. 2-1; two-way repeated-measures ANOVA with Tukey's multiple-comparisons test).

10.1523/ENEURO.0126-26.2026.f1-2 Figure 1-2 **MFB stimulation effectiveness variability amongst mice doesn’t affect performance in the Starmaze. A-B.** Place preference was confirmed by an increase in the time spent in the rewarded zone during the stimulation session on both day 1 and day 2 (Stim: average of the 30 µA stim trials, compared with the baseline session without stimulation (Pre, stim 0 µA) (Wilcoxon signed-rank test, *p* = 0.031 for both day 1 and day 2). Each color dot represents one mouse individual. **B-D.** Relationship between individual Starmaze performance and MFB stimulation effectiveness. MFB stimulation effectiveness was quantified for each mouse as the percentage increase in time spent in the rewarded zone between the Pre and Stim sessions of the place preference task. For Levels 1 and 2, Starmaze performance was quantified as the slope of the progression line between the two training days. For Level 3, performance was quantified as the percentage change in the number of direct trials from Day 4 to Day 7. **E-G.** Relationship between individual Starmaze mean percentage of direct trials and MFB stimulation effectiveness for level 1, 2 and 3. *R* indicates the Spearman correlation coefficient. Download Figure 1-2, TIF file.

**Figure 3.**
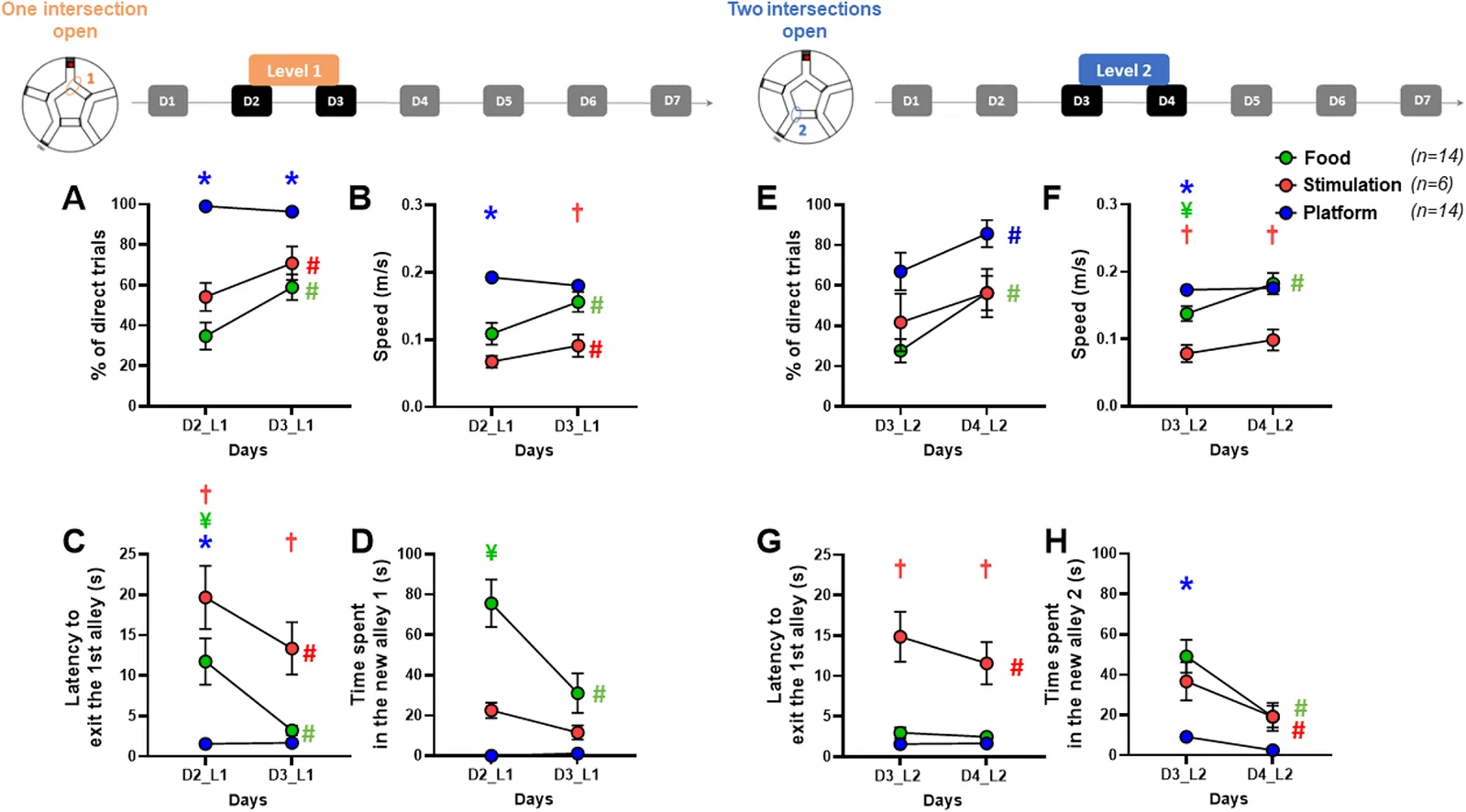
Goal-directed sequence with one decision point (Level 1, ***A–D***) or two decision points (Level 2, ***E–H***). This figure compares the performances of the three groups (Platform, Stimulation, and Food) in terms of (***A***,***E***) percentage of trials in which mice get directly to the reward; (***B***,***F***) the mean speed; (***C***,***G***) the latency to exit the departure alley; (***D***,***H***) the time spent in the newly opened alley, i.e., alley 1 for ***D*** and alley 2 for ***H***. *the platform group is different from the two other groups*.* ^†^the stimulation group is different from the two other groups. ^¥^the food group is different from the two other groups. *^#^*represents a significant day effect for the color-associated group (Extended Data Figs. 3-1, 3-2; 2-way repeated-measures ANOVA with Tukey's multiple-comparisons tests).

10.1523/ENEURO.0126-26.2026.f1-3 Figure 1-3 **Histological validation of MFB electrode implantation.** Representative coronal sections from the six mice of the MFB group (M1 to M6). White circles indicate electrode tip location. Sections were counterstained with DAPI. Download Figure 1-3, TIF file.

### Validation of MFB stimulation effectiveness

Before analyzing Starmaze performance, we verified the effectiveness of MFB stimulation using a conditioned place preference task (Extended Data Figs. 1-1, 1-2). Within a given day and stimulation intensity, behavioral responses were generally highly consistent between the two consecutive trials (Extended Data Fig. 1-1). Although the number of entries into the rewarded compartment showed some variability in a subset of animals, time spent in the rewarded compartment consistently confirmed the rewarding properties of MFB stimulation (Extended Data Fig. 1-2*A,B*). Interestingly, although the number of entries varied from Day 1 to Day 2, the time spent in the rewarded area remained unchanged. This suggests that the increased number of entries may partly reflect learning that repeated exits from and re-entries into the rewarded compartment trigger additional stimulation trains, rather than changes in the rewarding value of MFB stimulation itself. Finally, to determine whether variability in stimulation effectiveness could account for any behavioral performance observed during Starmaze learning, we performed correlational analyses between individual MFB effectiveness and Starmaze performance. No significant relationship was detected (Extended Data Fig. 1-2*C–H*).

#### Motivational and performance differences between aquatic and land-based groups in the absence of decision points

We first analyzed mice performances during the first 2 d of training, when the only available path in the maze led directly to the reward (Fig. 1). Under these conditions, with all intersections closed and no decision point, mice follow a unique route to get the reward. This phase allowed us to assess mice intrinsic motivation to get the reward without including any spatial processing or decision-making. Unexpectedly, MFB-rewarded mice showed slightly lower motivation compared with both the food- and platform-rewarded groups, as indicated by their longer latency to exit the start alley (Fig. 2*A*, two-way RM ANOVA, Day effect ns, Group effect *p* < 0.0001, Day × Group ns, post hoc Tukey's multiple-comparisons tests: Stimulation vs Platform, *p* < 0.0005, Stimulation vs Food, *p* = 0.06) and the lower percentage of rewards obtained on the first day (Fig. 2*B*, two-way RM ANOVA, Day effect ns, Group effect *p* < 0.005, Day × Group ns, post hoc Tukey's multiple-comparisons tests: Stimulation vs Platform, *p* < 0.0005, Stimulation vs Food, *p* *<* 0.05). Analysis of the distance traveled toward the reward and speed also revealed differences between the three groups of mice. The aquatic group showed a clear path optimization from Day 1 to Day 2, reaching the reward more rapidly. This improvement, reflected by both a reduction in distance (Fig. 2*C*, two-way RM ANOVA, Day effect *p* < 0.0005, Group effect *p* < 0.0005, Day × Group *p* < 0.0001, post hoc Tukey's multiple-comparisons tests: D1 vs D2, *p* < 0.0005, Tukey's multiple-comparisons test) and an increase in speed (Fig. 2*D*, two-way RM ANOVA, Day effect *p* < 0.005, Group effect *p* < 0.0001, Day × Group ns, post hoc Tukey's multiple-comparisons test: D1 vs D2, *p* < 0.005), was not observed in the other two groups (D1 vs D2, *p* > 0.05 for both groups and both parameters).

10.1523/ENEURO.0126-26.2026.f2-1 Figure 2-1 **Statistical analyses at Level 0 across the three reward conditions (MFB stimulation, food, and platform escape) for behavioral performances shown in Fig. 2.** The table reports 2-way repeated-measures ANOVAs (Group × Day) for four behavioral measures followed by Tukey’s post-hoc comparisons between groups for each testing day. For each contrast, q-values and adjusted P-values (Padj) are provided. Download Figure 2-1, TIF file.

In summary, at Level 0 of training, when no memory of the route is required, mice in the aquatic condition exhibited better performances from the very first day of training with a higher speed and a lower latency to exit the departure alley compared with both the MFB- and food-motivated groups.

### Increasing task difficulty reveals reward-specific patterns of navigation behavior

We next investigated the behavior of the mice when they were required to make one decision (turn left at the final junction to reach the reward; Fig. 3*A–D*) or two decisions (turn left at the first intersection and turn left again at the final junction; Fig. 3*E*–*H*). In such conditions, called Level 1 and Level 2, respectively, mice had to remember to turn left when a choice was required, both at the first and last turns.

10.1523/ENEURO.0126-26.2026.f3-1 Figure 3-1 **Statistical analyses at Level 1 across the three reward conditions (MFB stimulation, food, and platform escape) for behavioral performances shown in Fig. 3 A-D**. The table reports 2-way repeated-measures ANOVAs (Group × Day) for four behavioral measures followed by Tukey’s post-hoc comparisons between groups for each testing day. For each contrast, q-values and adjusted P-values (Padj) are provided. Download Figure 3-1, TIF file.

10.1523/ENEURO.0126-26.2026.f3-2 Figure 3-2 **Statistical analyses at Level 2 across the three reward conditions (MFB stimulation, food, and platform escape) for behavioral performances shown in Fig. 3 E-H**. The table reports 2-way repeated-measures ANOVAs (Group × Day) for four behavioral measures followed by Tukey’s post-hoc comparisons between groups for each testing day. For each contrast, q-values and adjusted P-values (Padj) are provided. Download Figure 3-2, TIF file.

The three groups of mice responded differently to these task demands. At Level 1, the aquatic-tested group used the optimal trajectory to the goal as soon as the first day of this new condition, outperforming the two land-based groups, as illustrated by the percentage of direct trials (Fig. 3*A*, two-way RM ANOVA, Day effect *p* < 0.0005, Group effect *p* < 0.0001, Day × Group *p* < 0.0005, post hoc Tukey's multiple-comparisons tests: D2_L1: Platform vs Stimulation, *p* < 0.0001, Platform vs Food, *p* < 0.0001) and speed (Fig. 3*B*, two-way RM ANOVA, Day effect *p* < 0.0005, Group effect *p* < 0.0001, Day × Group *p* < 0.0001, post hoc Tukey's multiple-comparisons tests: D2_L1: Platform vs Stimulation, *p* < 0.0001, Platform vs Food, *p* < 0.0001). Interestingly, although both MFB-stimulated and food-rewarded mice improved their trajectory over the two days of Level 1, they manifested different levels of motivation, as illustrated by the latency to start the task (Fig. 3*C*) and the exploratory behavior in the new alley of the Starmaze: while the MFB-stimulated mice stayed longer in the departure alley before starting the task on both days (Fig. 3*C*, two-way RM ANOVA, Day effect *p* < 0.0005, Group effect *p* < 0.0001, Day × Group *p* < 0.005, post hoc Tukey's multiple-comparisons tests: D2_L1: Stimulation vs Food, *p* < 0.05, Stimulation vs Platform, *p* < 0.0001, D3_L1: Stimulation vs Food, *p* < 0.005, Stimulation vs Platform, *p* < 0.005), the food-rewarded group spent more time in the new alley on the first day (Fig. 3*D*, two-way RM ANOVA, Day effect *p* < 0.005, Group effect *p* < 0.0001, Day × Group p < 0.005, post hoc Tukey's multiple-comparisons tests: D2_L1: Food vs Stimulation, *p* < 0.005, Food vs Platform, *p* < 0.0001) before reaching stimulated mice level on Day 2, remaining still higher than the aquatic group (Fig. 3*D*, post hoc Tukey's multiple-comparisons tests: D3_L1: Food vs Platform, *p* < 0.05).

The addition of another decision point at Level 2 induced contrasting results in the three conditions tested regarding mice capacity to adapt their trajectory. While the aquatic group performances were mildly affected by the presence of a second intersection, they remained at a superior level in terms of path optimization as shown by the increased percentage of direct trajectory (Fig. 3*E*, two-way RM ANOVA, Day effect *p* < 0.0005, Group effect *p* < 0.005, Day × Group ns, post hoc Tukey's multiple-comparisons tests: Platform: D3_L2 vs D4_L2, *p* < 0.05), highest speed (Fig. 3*F*, two-way RM ANOVA, Day effect *p* < 0.0005, Group effect *p* < 0.0001, Day × Group *p* < 0.005, post hoc Tukey's multiple-comparisons tests: D3_L2: Platform vs Stimulation, *p* < 0.0001, Platform vs Food, *p* < 0.05) and the lowest time spent in the new alley (Fig. 3*H*, two-way RM ANOVA, Day effect *p* < 0.05, Group effect *p* < 0.0001, Day × Group *p* < 0.05, post hoc Tukey's multiple-comparisons tests: D3_L2: Platform vs Stimulation, *p* < 0.05, Platform vs Food, *p* < 0.0001). Remarkably, the food tested mice showed a strong improvement in all the trajectory-associated parameters over the two days of Level 2 as reflected by the increased percentage of direct trials (Fig. 3*E*, Tukey's multiple-comparisons tests: Food: D3_L2 vs D4_L2, *p* < 0.0005), speed (Fig. 3*F*, Tukey's multiple-comparisons tests: Food: D3_L2 vs D4_L2, *p* < 0.0001), as well as the reduced time spent in the new alley (Fig. 3*H*, Tukey's multiple-comparisons tests: Food: D3_L2 vs D4_L2, *p* < 0.0001). On the other hand, MFB-stimulated mice did not significantly optimize their trajectory as reflected by the percentage of direct trials, even though they reduced their exploration of new peripheral alleys, as shown by the decrease in the time spent in the newly opened alley 2 (Fig. 3*H*, Tukey's multiple-comparisons tests: Stimulation: D3_L2 vs D4_L2, *p* < 0.05). While their latency to exit the first alley decreased between the days of Level 2 (Fig. 3*G*, Tukey's multiple-comparisons tests: D3_L2 vs D4_L2, *p* < 0.005), this motivation to initiate the task still remained lower than the two other groups (Fig. 3*G*, Tukey's multiple-comparisons tests: D3_L2 and D4_L2: Stimulation vs Platform, *p* < 0.0001, Stimulation vs Food, *p* < 0.0001).

### High cognitive demand equalizes performance through reward-dependent strategies

**Figure 4.**
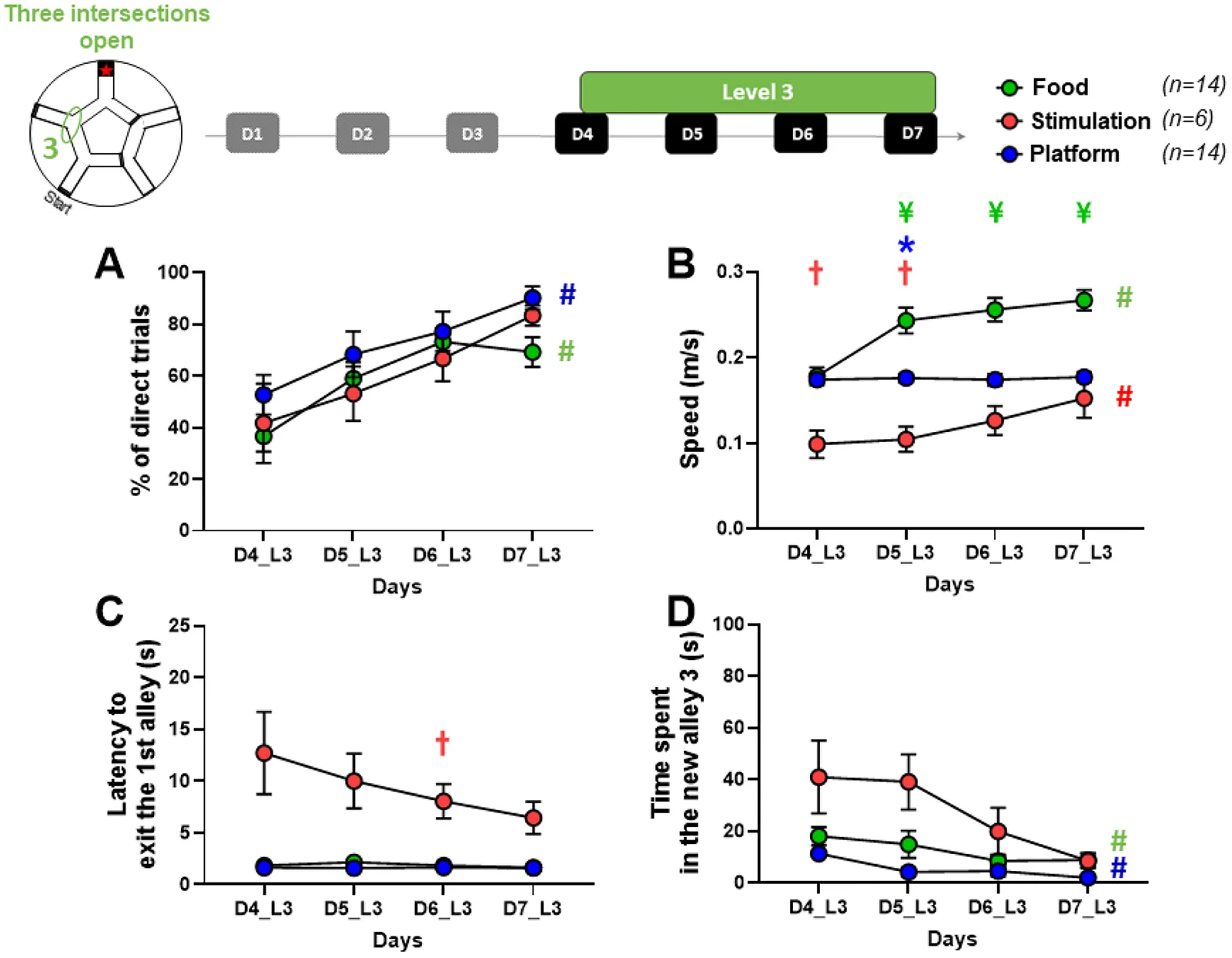
Level 3 (A 3-turn sequence to remember). This figure illustrates the percentage of direct trials (***A***), speed (***B***), latency to exit the first alley (***C***), and time spent in the newly opened arm (***D***) for Level (L) 3, where all peripheral alleys are open. The metrics are displayed across days (D4–D7) for three groups of mice receiving different types of rewards: food (green), MFB stimulation (red), and a platform for escaping water (blue).*the platform group is different from the two other groups. ^†^the stimulation group is different from the two other groups. ^¥^the food group is different from the two other groups. ^#^represents a significant difference between Day 4 and Day 7 for the color-associated group (Extended Data Fig. 4-1; two-way repeated-measures ANOVA with Tukey's multiple-comparisons tests).

Finally, we investigated mice performance dynamics at Level 3, when the cognitive demand increases to a full sequence of three consecutive correct turns to remember (left-right-left). Given the complexity of the task, we evaluated the performance across 4 d of training from Day 4 to Day 7 (Fig. 4). In this task level, the three groups showed equal ability to optimize their path to get the reward in terms of both performance quality and dynamics, as shown by the percentage of direct trials (Fig. 4*A*, two-way RM ANOVA, Day effect *p* < 0.0001, Group effect ns, Day × Group ns).

10.1523/ENEURO.0126-26.2026.f4-1 Figure 4-1 **Statistical analyses at Level 3 across the three reward conditions (MFB stimulation, food, and platform escape) for the behavioral parameters shown in Fig. 4.** The table reports 2-way repeated-measures ANOVAs (Group × Day) followed by Tukey’s post-hoc comparisons between groups for each testing day. For each contrast, q-values and adjusted P-values (Padj) are provided. Download Figure 4-1, TIF file.

To reach such performance levels, the three groups appeared to rely on different strategies, as reflected by changes in different behavioral parameters. Unlike the aquatic group, land-based tested mice (stimulation + food) showed an increase in speed over days (Fig. 4*B*, two-way RM ANOVA, Day effect *p* < 0.0001, Group effect *p* < 0.0001, Day × Group *p* < 0.0001, post hoc Tukey's multiple-comparisons tests: Food: D4_L3 vs D7_L3 *p* < 0.0001, Stimulation: D4_L3 vs D7_L3 *p* < 0.05), with the most pronounced improvement observed in the food-rewarded group, which reached a significantly higher speed than the other groups by the end of training (Fig. 4*B*, Tukey's multiple-comparisons tests: D7_L3: Food vs Stimulation, *p* < 0.005, Food vs Platform, *p* < 0.05). Interestingly, mice in the MFB group exhibited lower goal-directed motivation and a greater exploratory behavior upon exposure to novelty, as respectively indicated by the tendency to spend more time in the departure alley (Fig. 4*C*, two-way RM ANOVA, Day effect *p* < 0.0005, Group effect *p* < 0.0001, Day × Group *p* < 0.0001) and more time in the new alley at the onset of Level 3 (Fig. 4*D*, two-way RM ANOVA, Day effect *p* < 0.0001, Group effect *p* < 0.005, Day × Group *p* < 0.005).

Overall, as task complexity increased, the sharp distinction between aquatic and land-tested groups gave way to more subtle differences, with performance levels converging across all three groups. To further disentangle the strategies supporting this apparent convergence in performance, we next examined how trajectory optimization evolved on a finer timescale by analyzing distance traveled across sessions from Day 4 to Day 7.

### Trajectory optimization relies on reward-modulated memory dynamics

**Figure 5.**
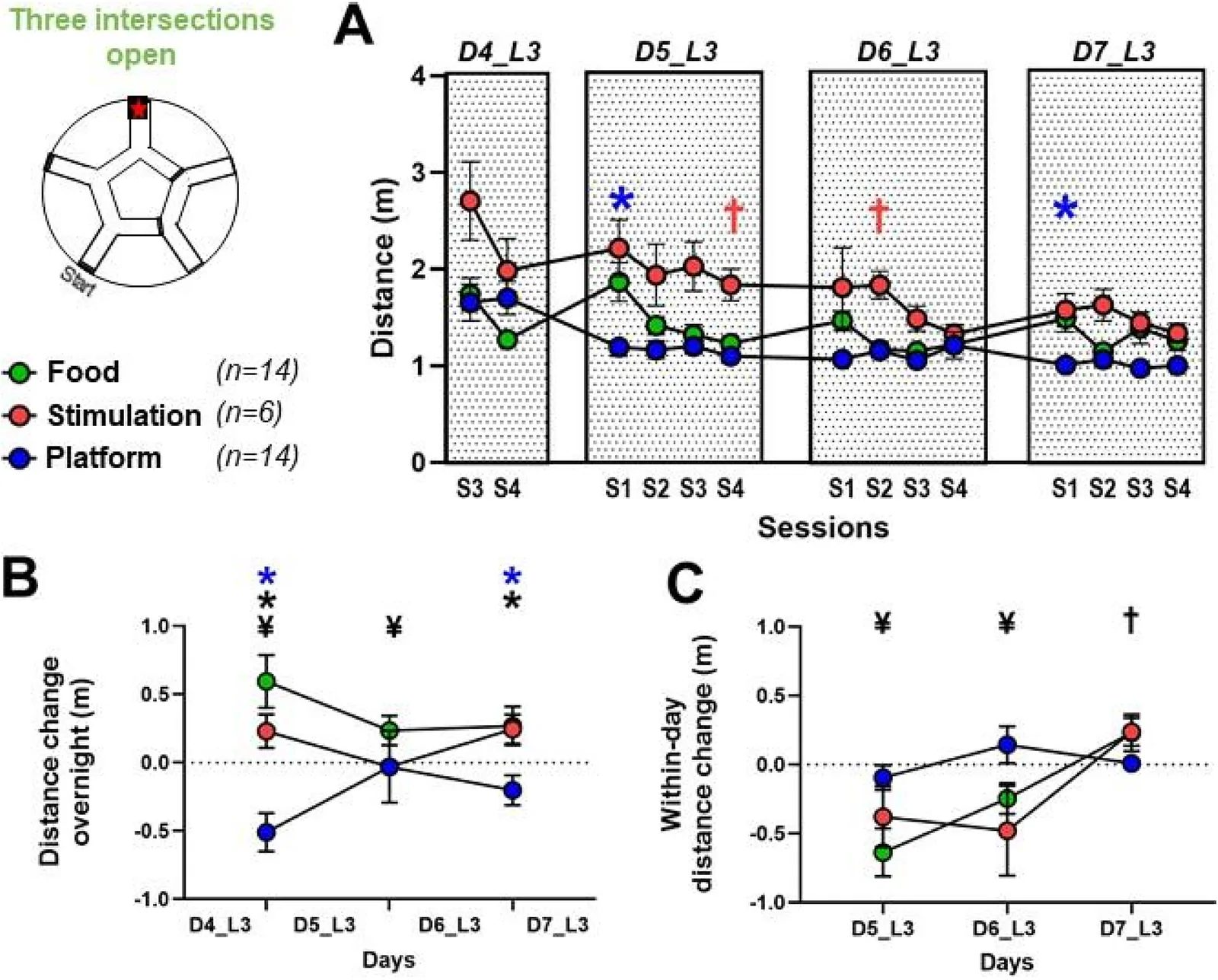
Dynamics of path optimization at Level 3. This figure illustrates the (***A***) distance, (***B***) distance difference between the last session of a day and the first session of the following day (S1n-S4n-1), and (***C***) difference between distance travelled at the first and last session of each day (S4-S1) of Level 3, where all peripheral alleys are open. The metrics are displayed across sessions (***A***) or days (***B***, ***C***; D4–D7) for three groups of mice receiving different types of rewards: food (green), MFB stimulation (red), and a platform for escaping water (blue). Negative values for ***B*** and ***C*** indicate a reduction in the distance between two consecutive sessions.*the aquatic group is different from the two other groups. ^†^the MFB group is different from the two other groups. ^¥^the food group is different from the two other groups (Extended Data Figs. 5-1, 5-2; 2-way repeated-measures ANOVA with Tukey's multiple-comparisons tests). Symbols in black indicate a score significantly different than 0 (one-sample Wilcoxon tests).

To further explore learning of a complex sequence in the three conditions tested, we used distance-based scores reflecting different mnesic components. First, we looked at memory consolidation overnight by comparing the distance animals traveled in the first session of a day with the last session of the previous day (Fig. 5*B*). Only the aquatic group showed efficient memory consolidation during Level 3 with a distance travelled during S1 of each day that was lower that during S4 of the previous day (Fig. 5*B*, Platform: *p* < 0.05 for D4_L3-D5_L3 and D6_L3-D7_L3, one-sample Wilcoxon test). In contrast, the land-based groups, especially those rewarded with food, consistently began each day by covering more distance than the previous one, although this difference gradually diminished over time (Fig. 5*B*, Food: *p* *<* 0.05 for D4_L3-D5_L3 and D5_L3-D6_L3, one-sample Wilcoxon test). Secondly, we measured memory within a day by comparing the distance traveled between the last and first session of each day (Fig. 5*C*). There were no clear changes in this parameter over days in the aquatic group. However, the food group showed a strong ability to correct their trajectory as reflected by the reduced distance during the last session compared with the first (Fig. 5*C*, Food: *p* < 0.05 for D5_L3 and D6_L3, one-sample Wilcoxon test). The MFB-stimulated group, on the other hand, showed improvement as seen by the difference in traveled distance between the last and first session of Day 5, with this gap decreasing over time and aligning with the aquatic group by Day 6 (Fig. 5*C*).

10.1523/ENEURO.0126-26.2026.f5-1 Figure 5-1 **Statistical analyses for travelled distances at Level 3 across the three reward conditions (MFB stimulation, food, and platform escape) for the travelled distance shown in Fig. 5A.** The table reports 2-way repeated-measures ANOVAs (Group × Day) for travelled distance followed by Tukey’s post-hoc comparisons between groups for each testing day. For each contrast, q-values and adjusted P-values (Padj) are provided. Download Figure 5-1, TIF file.

10.1523/ENEURO.0126-26.2026.f5-2 Figure 5-2 **Statistical analyses at Level 3 across the three reward conditions (MFB stimulation, food, and platform escape) for the behavioral parameters shown in Fig. 5 B-C.** The table reports 2-way repeated-measures ANOVAs (Group × Day) followed by Tukey’s post-hoc comparisons between groups for each testing day. For each contrast, q-values and adjusted P-values (Padj) are provided. Download Figure 5-2, TIF file.

In summary, although overall performance levels were similar between the three groups, trajectory analyses on a finer scale revealed distinct dynamics during learning of a complex three-turn sequence: aquatic-tested mice rapidly optimized their path and maintained stable performance, food-rewarded mice showed fluctuating trajectories and within-day correction, and MFB-stimulated mice exhibited a gradual and delayed improvement.

## Discussion

Navigation emerges from interactions between memory systems and reward-based processes (Packard and McGaugh, 1996; Schultz et al., 1997; Sosa and Giocomo, 2021). In this study, we compared the effects of three distinct reward modalities on the acquisition of a sequential navigation task in the Starmaze, while progressively increasing task complexity. Incremental training protocols facilitate spatial learning by progressively structuring behavior while maintaining engagement (Holleman et al., 2019). This design allowed us to examine how motivational context interacts with cognitive demand to shape learning dynamics and behavioral strategies.

### Low task complexity: motivational bias and early strategy selection

At low levels of difficulty (Levels 0–1), performance differences primarily reflected motivational context rather than cognitive demand. At this stage, navigation can rely on simple or habitual responses, with no need for sequential memory processing (Packard and McGaugh, 1996; Yin and Knowlton, 2006). Mice in the aquatic condition rapidly reached ceiling performance, suggesting strong engagement and rapid reliance on automatized strategies. Aversive contexts are known to enhance learning speed and bias behavior toward efficient exploitation (McGaugh, 2004). In contrast, animals in land-based conditions showed increased exploration, likely reflecting adaptive information-gathering strategies rather than impaired learning. This behavior is consistent with theoretical frameworks describing the exploration–exploitation trade-off in reinforcement learning (Daw et al., 2006; Cohen and Ranganath, 2007) and with recent integrative views of navigation as a reward-driven process (Sosa and Giocomo, 2021).

Because groups differed in baseline motivational states, comparisons at this stage do not isolate learning mechanisms per se. Instead, these findings highlight how reward modality biases early strategy selection under low cognitive demand.

### Intermediate complexity: dissociation between performance and strategy

At intermediate complexity (Level 2), all groups achieved similar performance levels but exhibited distinct behavioral strategies, indicating that reward modality influences how the task is solved rather than whether it can be solved. MFB-stimulated mice showed longer initiation latencies and reduced speed. Natural rewards such as food or escape involve multisensory and physiological signals that enhance motivation and facilitate action initiation (Berridge and Robinson, 1998; Burnett et al., 2016). Hence, Burnett et al. (2016) demonstrated that physiological hunger suppresses competing motivational drives, supporting the idea that food reward can promote efficient spatial behavior. In contrast, MFB stimulation directly activates reward circuits without physiological feedback, potentially increasing the computational demands required for action–outcome association (Shizgal, 1997; Schultz, 2015). Recent work further supports the idea of a crucial temporal coordination between the rule representation in mPFC/CA1, the dopamine reward signal, and behavioral strategy (Ding et al., 2025). Thus, even when performance is matched, reward modality shapes underlying decision-making strategies.

### High complexity: convergence of performance, divergence of learning strategy

At the highest level of complexity (Level 3), all groups converged toward similar performance levels, enabling direct comparison under matched cognitive demands. Despite similar outcomes, detailed behavioral analyses revealed distinct learning strategies associated with each reward modality. In the aquatic condition, mice rapidly reduced exploratory behavior and showed progressive optimization of trajectories across days consistent with a consolidation process (Dudai et al., 2015; Klinzing et al., 2019). This is coherent with previous studies suggesting that sequence-based behavior in aquatic conditions implies hippocampal-dependent processes relying on model-free reinforcement learning associated with a memory of past actions (Babayan et al., 2017). Food-rewarded animals displayed within-session improvements but reduced stability across days, suggesting greater reliance on short-term or working memory processes. This is consistent with hippocampal–prefrontal interactions supporting flexible decision-making and ongoing evaluation (Euston et al., 2012; Spellman et al., 2015). Recent studies highlight the critical role of dopamine in modulating hippocampal activity and spatial behavior (Baeuchl et al., 2023) and in shaping synaptic plasticity underlying contextual learning (Sayegh et al., 2024). However, in contrast to the two other groups, MFB-stimulated mice showed slower and more gradual learning, without clear consolidation effects, alongside higher variability which cannot be attributed to differences in stimulation effectiveness (Extended Data Fig. 1-2). These findings suggest that as task complexity increases, the influence of rewards driven by physiological factors becomes more critical in consolidating sequence-based behavior than the activation of the reward circuit per se.

### Reward modality and neural systems underlying navigation

Together, these findings support the idea that identical navigational demands can be solved using distinct strategies. Spatial navigation depends on interactions between hippocampal and striatal circuits supporting flexible and habitual behavior (Packard and McGaugh, 1996; Graybiel, 2008). In the aquatic condition, aversive motivation may promote rapid engagement of hippocampal circuits and facilitate consolidation. Food reward appears to favor flexible, exploratory strategies potentially involving hippocampal–prefrontal networks. In contrast, MFB stimulation likely engages dopaminergic circuits involved in valuation and planning (Wise, 2004; Ilango et al., 2014). Recent work further emphasizes that hippocampal dopamine signaling is not homogeneous but organized into distinct functional pathways that differentially contribute to learning and navigation (Heer and Sheffield, 2024) and that dopamine broadly regulates hippocampal-dependent memory (Tsetsenis et al., 2021). These findings reinforce the view that navigation arises from tight integration between spatial memory systems and reward-processing circuits (Kesner et al., 2022). Importantly, while MFB stimulation is known to sustain high levels of performance and stable motivation in perceptual tasks (Verdier et al., 2022), it did not enhance learning in our sequential navigation paradigm. This dissociation suggests that the efficiency of MFB reinforcement may depend on task structure and that direct activation of reward circuits is not sufficient to support optimal learning when behavior relies on sequential decision-making.

### Conclusion

Together, our results show that reward modality does not simply influence the rate of learning but shapes the behavioral strategies used to solve a sequential navigation task. While all groups reached comparable levels of performance under increasing task demands, they relied on distinct dynamics of exploration, action initiation, and consolidation. Notably, despite its well-established ability to sustain high performance in other behavioral paradigms, MFB stimulation did not confer any advantage in this context and was associated with slower and less consolidated learning. This highlights that the impact of reward signals on behavior critically depends on the nature of the task and that direct activation of reward circuits is not sufficient to support efficient learning in a sequential goal-directed navigation task.

## Synthesis

Reviewing Editor: Michael McDannald, Boston College Decisions are customarily a result of the Reviewing Editor and the peer reviewers coming together and discussing their recommendations until a consensus is reached. When revisions are invited, a fact-based synthesis statement explaining their decision and outlining what is needed to prepare a revision will be listed below. The following reviewer(s) agreed to reveal their identity: Jerome Epsztein.

This manuscript investigates how reward modality and task complexity jointly shape learning dynamics in a sequential spatial navigation task. Using three groups of mice rewarded with platform escape (aquatic maze), food pellets, or medial forebrain bundle (MFB) electrical stimulation, the authors systematically varied the number of decision points across training (0-3 intersections). The central findings are that aquatic reward confers an early performance advantage at low task complexity, that all groups converge toward comparable performance at the highest complexity level, and that the strategies underlying this convergence diverge across reward conditions - as revealed by fine-scale trajectory and latency analyses. The establishment of a dry-land version of the Starmaze compatible with electrophysiological recording is also noted as a methodological contribution to the field.

The reviewers are positively inclined toward the manuscript and agree that it represents a meaningful contribution. However, a primary concern centers on the functional validation of the MFB electrode implantation and its relationship to behavioral outcomes. The place preference data in Supplemental Figure 1 reveal considerable inter-session and inter-individual variability in the rewarding effectiveness of MFB stimulation: for several mice (e.g., M1, M4, M6), the two validation sessions yield markedly discrepant results. The authors should clearly label which data points correspond to the first versus second session in Supplemental Figure 1, and discuss whether this variability is expected - for instance, whether it may reflect learning of the location-stimulation association across sessions. Critically, the reviewers agree that the authors should go beyond discussion and provide a correlational analysis between each animal's MFB effectiveness (e.g., percent increase in rewarded zone entries relative to the 0 µA baseline) and its maze performance across training levels. This analysis would help determine whether individual differences in stimulation efficacy account for the lower and more variable performance observed in the MFB group.

Two additional major points were raised. First, the behavioral measure of time spent in the newly opened alley requires clarification: the manuscript should specify which alley is designated "new" at each complexity level, and provide a more explicit interpretive framework for this measure - particularly with respect to the exploration-exploitation tradeoff and how reward modality is expected to modulate it. The trends observed across levels in the food and MFB groups (including the day-to-day decrease in the food group and the persistently elevated values in the MFB group) warrant dedicated discussion. Second, the description of trajectory optimization in the MFB group at Level 2 (page 15, Figure 3H) requires clarification: the text states that MFB mice do not improve their trajectories, yet a p-value below 0.05 is reported; this apparent inconsistency should be resolved. More broadly, the figures would benefit from the addition of significance indicators for within-group day-to-day comparisons, to spare readers from cross-referencing the text and supplementary tables.

Several minor points should also be addressed in revision. The significance statement's use of "sequential demands" should be defined explicitly. The reference to European Council Directive 86/809/EEC in the Methods should be updated to Directive 2010/63/EU. The x-axis inconsistency between panels A and E of Figure 3 should be corrected. Two instances of imprecise phrasing on pages 13 and 16 should be revised as suggested. Taking these points together, the reviewers recommend a revise and resubmit, with the correlational analysis of MFB stimulation efficacy and maze performance representing the key new element requested.

Reviewer comments:

In this article, the authors investigate whether and how different rewards influence learning in a spatial memory task based on sequence learning: the star maze. The authors compare learning among mice rewarded with the possibility to climb onto a platform in an aquatic version of the maze, with food in a dry version of the maze for food-deprived animals, and with Medial Forebrain Bundle stimulation, which directly activates among others reward-related efferents of the dopaminergic system. They use different versions of the maze of increasing complexity, starting with the simplest version in which the animals have no choice but to follow the path leading to the reward, to increasingly complicated versions where the animals must recall behavioral sequences requiring them to make one (level 1), two (level 2) or three (level 3) correct decisions (each decision being to turn right or left) to reach the reward. The authors demonstrate that different rewards modify learning, notably with a very significant effect of the platform in the aquatic maze, associated with the strongest motivation. In contrast, MFB stimulation leads to poorer performance and appears to be associated with lower motivation, with a longer latency to leave the first arm to initiate locomotor activity and slower speed in the maze. Interestingly, in the most complex version of the task requiring three decision-making steps, performance in the aquatic maze is no better than in the other conditions, although behavioral differences indicate that motivational differences are still present (particularly regarding MFB stimulation, which is consistently associated with slower running speeds and a longer latency to engage in exploratory activity). A more detailed analysis of the trajectories suggests that even if performance is not significantly different, the strategies used to complete the task may vary depending on the type of reward.

In summary, to the best of my knowledge, this study is the first to test the effect of different rewards on learning. In most studies, a single reward type is tested, sometimes with varying amounts, and the conclusions are often generalized. This study, on the contrary, shows that the type of reward influences both performance and the strategies used to solve a task and thus represents an important contribution to the field. This study also establishes the conditions for performing the dry-land version of the star maze task, opening the possibility of combining this task with electrophysiological recordings that are difficult to combine with aquatic mazes.

Key points:

1- MFB stimulation is associated with a lower motivation to perform the task. An important question concerns its effectiveness. This is tested using a place preference protocol, but only two sessions were conducted, and these two sessions often yield very different results. For example, for Mouse 1, stimulation appears to have no effect on behavior in one session (number of entries similar to the session without stimulation), whereas the other session shows an effect (increase in the number of entries depending on stimulation intensity); see Figure S1. This is also the case for Mouse 4 and, to some extent, Mouse 6 (for 20 µA). Is this effect expected? Could you label the dots corresponding to the first and second session in Fig S1? Is this effect related to learning the association between the rewarding effect of the stimulation and the location associated with that stimulation? This point deserves discussion in the manuscript.

2- While some behavioral parameters are easy to interpret, others are more complex, such as the time spent in the new alley. It would be helpful to specify which alley is new in the different versions of the task. At the end of the manuscript, it is stated that this time is associated with exploratory behavior related to novelty (p. 17). However, the way in which the reward might influence this exploratory behavior is not easy to interpret. Is this time related to the exploitation/exploration tradeoff? How should we interpret the observed trend across levels of complexity in the food reward condition (with a steady decrease over sessions with a new alley on the first day the alley being less new on the second day) and the MFB stimulation condition?

3- Regarding the optimization of trajectories measured by the number of direct paths in the MFB group (p15 and Figure 3H), the text mentions that they do not improve the trajectories, yet the p value is < 0.05? A clearer visualization of the significance of differences between days in the figures would be helpful (see minor points).

Minor points:

1- Examining the figures allows one to visualize the significant differences between the various rewards on a given day but does not allow one to quickly see if there is a significant change between two days (no asterisks on the graph). This indication would be useful to prevent the reader from having to look up p values in the text or table.

2- In the significance statement: "as sequential demands increase." Could you clarify what "sequential demands" means?

3- Materials and Methods: "European Council Directive (86/809/EEC)." The new directive currently in use is Directive 2010/63/EU.

4- Figure 3: The x-axis in Panel A differs from that in Panel E

5- P16: "as reflected by changes in other behavioral parameters" could be "as reflected by changes in different behavioral parameters".

6- P13: "this cognitive demand was supported differently": it is unclear to me what "supporting a cognitive demand" means.

## Author Response

We thank the reviewers for the constructive comments on the manuscript and the editor for the helpful synthesis of the reviews. We have carefully addressed all reviewer comments and editor recommendations, as detailed in the point-by-point responses below.

Manuscript Instructions - Please remove the author institution from the Methods section. In order to facilitate the double-anonymous review process, no information may be present that will reveal the identity of any author. If you feel this information is essential to the review of the manuscript, then please reply to this email to inquire whether your manuscript may proceed with a non-double-anonymous review process.

We have now removed the author institution from the method section (Page 5).

- Supplemental Figure 1 and Supplemental Table 2 were provided but were not cited in the text. Please either add the reference(s) or remove the figure.

Following journal's figure and table formatting guidelines as well as reviewer's suggestions (see below), we had to reorganize the supplementary figures and tables. We now have 3 supplementary figures as well as 6 supplementary tables that are all cited in the main text or figure legends (see legends of fig 2, fig 3, fig. 4 and fig 5 as well as pages 8-10).

--------------------------------------------- Synthesis Statement for Author (Required):

This manuscript investigates how reward modality and task complexity jointly shape learning dynamics in a sequential spatial navigation task. Using three groups of mice rewarded with platform escape (aquatic maze), food pellets, or medial forebrain bundle (MFB) electrical stimulation, the authors systematically varied the number of decision points across training (0-3 intersections). The central findings are that aquatic reward confers an early performance advantage at low task complexity, that all groups converge toward comparable performance at the highest complexity level, and that the strategies underlying this convergence diverge across reward conditions - as revealed by fine-scale trajectory and latency analyses. The establishment of a dry-land version of the Starmaze compatible with electrophysiological recording is also noted as a methodological contribution to the field.

The reviewers are positively inclined toward the manuscript and agree that it represents a meaningful contribution. However, a primary concern centers on the functional validation of the MFB electrode implantation and its relationship to behavioral outcomes. The place preference data in Supplemental Figure 1 reveal considerable inter- and inter-individual variability in the rewarding effectiveness of MFB stimulation: for several mice (e.g., M1, M4, M6), the two validation sessions yield markedly discrepant results. The authors should clearly label which data points correspond to the first versus second session in Supplemental Figure 1, and discuss whether this variability is expected - for instance, whether it may reflect learning of the location-stimulation association across sessions.

We thank the reviewers for this constructive suggestion. We have now labeled each data point in Supplementary Figure 1 to indicate whether it corresponds to the first or the second place-preference trial for both Day 1 and Day 2. This shows that, within a given day and stimulation intensity, behavioral responses were generally highly consistent between the two consecutive trials. The largest differences were observed between Day 1 and Day 2 rather than between successive trials within the same day. The increase in the number of entries from day 1 to day 2 was expected given the rule associated with reward delivery. As stimulation was delivered upon each entry into the rewarded zone, animals had to leave and re-enter the compartment to trigger additional stimulation trains. Accordingly, the increase in the number of entries between day 1 and day 2 may reflect learning that each exit and re-entry into the rewarded compartment triggers a new stimulation train. To confirm this, we performed complementary analyses based on the time spent in the rewarded zone, a parameter commonly used in studies employing MFB stimulation (see for example, Verdier et al., 2022) that does not depend on this leave-and-re-enter requirement. Interestingly, we did not observe an increase in this parameter between day 1 and day 2. This dissociation suggests that the increase in entry number is unlikely to reflect a stronger rewarding effect of MFB stimulation across sessions. Instead, it most likely reflects learning of the reward rule, whereby animals must leave and re-enter the rewarded zone to obtain additional stimulation trains. We have added this point to the manuscript (see pages 6 and 9-10).

Critically, the reviewers agree that the authors should go beyond discussion and provide a correlational analysis between each animal's MFB effectiveness (e.g., percent increase in rewarded zone entries relative to the 0 µA baseline) and its maze performance across training levels. This analysis would help determine whether individual differences in stimulation efficacy account for the lower and more variable performance observed in the MFB group.

We agree with the reviewers and have now performed the requested correlational analyses between individual MFB stimulation effectiveness and two different parameters reflecting Starmaze performances. As shown in new Supplementary Figure 2, MFB stimulation was efficient for all mice (see panel A and B) with diverse degree of efficacy but no significant correlation was detected between MFB stimulation effectiveness and learning performance at any level of the Starmaze task (see panel C-H), indicating that inter-individual differences in stimulation efficacy cannot explain the lower or more variable performance observed in the MFB group (see pages 9-10).

Two additional major points were raised. First, the behavioral measure of time spent in the newly opened alley requires clarification: the manuscript should specify which alley is designated "new" at each complexity level, and provide a more explicit interpretive framework for this measure - particularly with respect to the exploration-exploitation tradeoff and how reward modality is expected to modulate it. The trends observed across levels in the food and MFB groups (including the day-to-day decrease in the food group and the persistently elevated values in the MFB group) warrant dedicated discussion.

We thank the reviewers for pointing out this lack of clarity. Indeed, this parameter specifically reflects exploratory behavior toward novelty and can be viewed as an indicator of the exploration component of the exploration-exploitation tradeoff. During Level 1, it corresponds to the time spent exploring Alley 1, which is newly accessible at this stage of the task. During Level 2, it corresponds exclusively to the exploration of Alley 2, as Alley 1 is no longer novel, having already been accessible during Level 1. We have now clarified this point in the axis labels and figure legend of Figures 3D and 3H as well as in the text (page 13).

Second, the description of trajectory optimization in the MFB group at Level 2 (page 15, Figure 3H) requires clarification: the text states that MFB mice do not improve their trajectories, yet a p-value below 0.05 is reported; this apparent inconsistency should be resolved. More broadly, the figures would benefit from the addition of significance indicators for within-group day-to-day comparisons, to spare readers from cross-referencing the text and supplementary tables.

We agree with the reviewer and have now included an indication of significance between days on the figure 2 (see answer to minor points).

Several minor points should also be addressed in revision.

The significance statement's use of "sequential demands" should be defined explicitly.

We agree that this wording was ambiguous and have replaced it by "as the number of successive choices increase" (page 2).

The reference to European Council Directive 86/809/EEC in the Methods should be updated to Directive 2010/63/EU.

This reference is now updated (See page 5) The x-axis inconsistency between panels A and E of Figure 3 should be corrected.

We assume the reviewer refers to the different x-axis labels between panels A-D and E-H. These panels correspond to different task levels (Level 1 versus Level 2) and therefore display different session labels. To avoid any ambiguity, we have clarified this point in the figure legend and in the main text (page 13) Two instances of imprecise phrasing on pages 13 and 16 should be revised as suggested. Taking these points together, the reviewers recommend a revise and resubmit, with the correlational analysis of MFB stimulation efficacy and maze performance representing the key new element requested.

We have corrected the imprecise phrasing highlighted by the reviewers (see our response to reviewers below) and resubmit now a full revised version including the correlational analysis of MFB stimulation efficacy and maze performance (Supp Fig 1 and Supp Fig 2).

Reviewer comments:

In this article, the authors investigate whether and how different rewards influence learning in a spatial memory task based on sequence learning: the star maze. The authors compare learning among mice rewarded with the possibility to climb onto a platform in an aquatic version of the maze, with food in a dry version of the maze for food-deprived animals, and with Medial Forebrain Bundle stimulation, which directly activates among others reward-related efferents of the dopaminergic system. They use different versions of the maze of increasing complexity, starting with the simplest version in which the animals have no choice but to follow the path leading to the reward, to increasingly complicated versions where the animals must recall behavioral sequences requiring them to make one (level 1), two (level 2) or three (level 3) correct decisions (each decision being to turn right or left) to reach the reward. The authors demonstrate that different rewards modify learning, notably with a very significant effect of the platform in the aquatic maze, associated with the strongest motivation. In contrast, MFB stimulation leads to poorer performance and appears to be associated with lower motivation, with a longer latency to leave the first arm to initiate locomotor activity and slower speed in the maze. Interestingly, in the most complex version of the task requiring three decision-making steps, performance in the aquatic maze is no better than in the other conditions, although behavioral differences indicate that motivational differences are still present (particularly regarding MFB stimulation, which is consistently associated with slower running speeds and a longer latency to engage in exploratory activity). A more detailed analysis of the trajectories suggests that even if performance is not significantly different, the strategies used to complete the task may vary depending on the type of reward.

In summary, to the best of my knowledge, this study is the first to test the effect of different rewards on learning. In most studies, a single reward type is tested, sometimes with varying amounts, and the conclusions are often generalized. This study, on the contrary, shows that the type of reward influences both performance and the strategies used to solve a task and thus represents an important contribution to the field. This study also establishes the conditions for performing the dry-land version of the star maze task, opening the possibility of combining this task with electrophysiological recordings that are difficult to combine with aquatic mazes.

Key points:

1- MFB stimulation is associated with a lower motivation to perform the task. An important question concerns its effectiveness. This is tested using a place preference protocol, but only two sessions were conducted, and these two sessions often yield very different results. For example, for Mouse 1, stimulation appears to have no effect on behavior in one session (number of entries similar to the session without stimulation), whereas the other session shows an effect (increase in the number of entries depending on stimulation intensity); see Figure S1. This is also the case for Mouse 4 and, to some extent, Mouse 6 (for 20 µA). Is this effect expected? Could you label the dots corresponding to the first and second session in Fig S1? Is this effect related to learning the association between the rewarding effect of the stimulation and the location associated with that stimulation? This point deserves discussion in the manuscript.

We thank the reviewer for this constructive suggestion. We have performed 2 trials per day during two consecutive days. We have now labeled each data point in supplementary Figure 1 to clarify the data coming from the first and second trials on the first and second days of place preference testing. In addition, we have included complementary analyses assessing also the time spent in the rewarded area, a parameter commonly used in studies employing MFB stimulation (see for example, Verdier et al., 2022). We have added this information in the method (page 6), and the supplementary Figure 1. Interestingly, although several animals displayed an increase in the number of entries into the rewarded area from Day 1 to Day 2, this effect was not paralleled by a corresponding increase in the time spent in the rewarded compartment. This dissociation suggests that the higher number of entries may not necessarily reflect a stronger place preference. Instead, it may partly reflect learning that repeated exits and re-entries into the rewarded compartment trigger additional stimulation trains. However, because this increase in entries was not accompanied by a corresponding increase in time spent in the rewarded compartment, it is unlikely to reflect a change in the rewarding value of MFB stimulation from day 1 to day 2. We have added this point to the manuscript (see dedicated section in the results pages 9-10). Importantly, all animals consistently increase the time spent in the rewarded area under stimulation compared to pre stim condition (Supp Fig 2A and 2B) supporting the rewarding nature of the MFB stimulation.

2- While some behavioral parameters are easy to interpret, others are more complex, such as the time spent in the new alley. It would be helpful to specify which alley is new in the different versions of the task. At the end of the manuscript, it is stated that this time is associated with exploratory behavior related to novelty (p. 17). However, the way in which the reward might influence this exploratory behavior is not easy to interpret. Is this time related to the exploitation/exploration tradeoff? How should we interpret the observed trend across levels of complexity in the food reward condition (with a steady decrease over sessions with a new alley on the first day the alley being less new on the second day) and the MFB stimulation condition? We thank the reviewer for pointing out this lack of clarity. Indeed, this parameter specifically reflects exploratory behavior toward novelty and can be viewed as an indicator of the exploration component of the exploration-exploitation tradeoff. During Level 1, it corresponds to the time spent exploring Alley 1, which is newly accessible at this stage of the task. During Level 2, it corresponds exclusively to the exploration of Alley 2, as Alley 1 is no longer novel, having already been accessible during Level 1. We have now clarified this point in the axis labels and figure legend of Figures 3D and 3H.

3- Regarding the optimization of trajectories measured by the number of direct paths in the MFB group (p15 and Figure 3H), the text mentions that they do not improve the trajectories, yet the p value is < 0.05? A clearer visualization of the significance of differences between days in the figures would be helpful (see minor points).

We agree with the reviewer and have now included an indication of significance between days on the Figure 2 (see our response to the minor points).

Minor points:

1- Examining the figures allows one to visualize the significant differences between the various rewards on a given day but does not allow one to quickly see if there is a significant change between two days (no asterisks on the graph). This indication would be useful to prevent the reader from having to look up p values in the text or table.

We thank the reviewer for this remark. We have now added an indication of significant change between two days directly on the graph (symbol #, also explained in the legend) 2- In the significance statement: "as sequential demands increase." Could you clarify what "sequential demands" means? We agree that this wording was ambiguous and have replaced it by "as the number of successive choices increase" (page 2).

3- Materials and Methods: "European Council Directive (86/809/EEC)." The new directive currently in use is Directive 2010/63/EU.

We thank the reviewer for this clarification, the correct statement is now included (see page 5).

4- Figure 3: The x-axis in Panel A differs from that in Panel E We assume the reviewer refers to the different x-axis labels between panels A-D and E-H. These panels correspond to different task levels (Level 1 versus Level 2) and therefore display different session labels. To avoid any ambiguity, we have clarified this point in the figure legend and in the main text (see page 13).

5- P16: "as reflected by changes in other behavioral parameters" could be "as reflected by changes in different behavioral parameters".

We have modified the manuscript accordingly (see page 14).

6- P13: "this cognitive demand was supported differently": it is unclear to me what "supporting a cognitive demand" means.

This phrasing is now replaced by "the three groups of mice responded differently to these task demands" (see page 15)
